# Impact of 8 Weeks of Moderate- Versus High-Intensity Interval Training on Sleep Quality

**DOI:** 10.3390/ijerph23020202

**Published:** 2026-02-04

**Authors:** Jean Bourgeois, Charlotte Domange, Bert Celie

**Affiliations:** Research Unit in Cardio-Respiratory Physiology, Exercise & Nutrition, Faculty of Human Movement Sciences, Université Libre de Bruxelles, 1070 Brussels, Belgium

**Keywords:** sleep quality, high-intensity interval training (HIIT), moderate-intensity continuous training (MICT), Pittsburgh sleep quality index (PSQI), heart rates (HR)

## Abstract

Sleep is a fundamental aspect of physiological and psychological functioning, and the beneficial effect of exercise on sleep quality and quantity, depending on training modality, remains underexplored. This study compared the effects of high-intensity interval training (HIIT) versus a moderate-intensity continuous training (MICT) regime on sleep quality. Twenty-five participants (sixteen men, nine women) were randomly assigned to 8-week HIIT or MICT programs. Anthropometric data, blood pressure, and maximal exercise tests (VO_2max_, lactate, heart rate) were conducted one week before and after training. Sleep quality was evaluated daily through self-reported perception and duration and via the Pittsburgh Sleep Quality Index (PSQI) at baseline, one week, and two weeks post-intervention. Data were analyzed in SPSS version 29 using repeated-measures ANOVA. PSQI scores improved significantly over time (*p* = 0.013), regardless of modality, with no significant group or interaction effects. Cardiorespiratory fitness improved for all participants, with significant gains in VO_2max_ (*p* = 0.009), maximal aerobic speed (*p* < 0.001), and reduced maximal heart rate (HIIT: *p* = 0.003; MICT: *p* = 0.021). Sleep perception showed no significant change during training (*p* = 0.063), with a slight improvement trend. In conclusion, exercise training improves sleep quality regardless of modality. Running three sessions per week for eight weeks enhances both aerobic and cardiorespiratory fitness, along with sleep quality. Physical activity is therefore an effective non-pharmacological strategy to improve sleep.

## 1. Introduction

Sleep is fundamental to physiological and psychological balance and is recognized as one of the three essential pillars of health, alongside nutrition and physical activity [1]. Sleep deprivation or poor sleep quality is a widespread issue, frequently reported across populations and increasingly regarded as a societal concern. However, its health consequences are not widely recognized, and many medical school curricula do not emphasize the importance of behavioral factors in overall health [2,3]. Sleep quality is affected by various factors, including circadian rhythm disruptions, genetics, diet, stress, and socioeconomic conditions [3].

Given the consequences of sleep deprivation, promoting qualitative and sufficient sleep is essential. A healthy diet, reduced stimulant intake, and regular physical activity are recommended strategies [4,5]. It is well known that a balanced combination of physical activity and qualitative sleep yields positive outcomes across physical, cognitive, and social domains [6]. Exercise has been shown to enhance sleep quality both subjectively and objectively [7]. Numerous studies highlight the benefits of moderate physical activity on sleep across diverse populations, even without specific training protocols [8,9,10,11]. Hence, meta-analytical data reports an improvement in sleep quality based on PSQI scores, indicating that both physical exercise and mind–body practices (such as yoga and tai chi) can enhance sleep quality [12]. However, no specific type of exercise, whether physical or mind–body, was found to be clearly superior in addressing sleep problems. Physical and mental exercise may improve sleep by strengthening parasympathetic nervous system activity at rest and promoting emotional release, thereby supporting the regulation of awareness and attention [6]. A recent meta-analysis by De Nys et al. [13] further examined the impact of physical exercise on cortisol regulation and sleep quality. Their findings indicate that light-to moderate-intensity activities, whether aerobic or mind–body-based, help modulate stress and improve sleep. This hormonal regulation appears to be a key factor in enhancing overall sleep quality and underscores the relevance of exploring potential correlations between athletic performance and sleep quality.

Targeted research into the types of exercise most beneficial for sleep quality could lead to more precise recommendations. More specifically, little is known in the scientific literature about the impact of different exercise modalities on sleep quality and time. Accordingly, this study investigates the impact of different running training regimens (endurance-based versus high-intensity interval training) on sleep quality in a healthy, non-athletic population. It aims to determine which modality is more conducive to improving sleep, addressing the following central question: what is the effect of different running training regimes on sleep quality in healthy, non-athletic individuals?

## 2. Materials and Methods

### 2.1. Population

Thirty-two individuals participated in the study, including eleven women and twenty-one men, recruited through word of mouth and social media platforms. Eligibility criteria required subjects to be between 18 and 45 years old, to have a body mass index (BMI) between 18 and 30, to be either beginners in sports or sedentary, not to have professional or personal obligations interfering with the sleep cycle, to consume fewer than 10 alcoholic drinks per week, to be free of chronic diseases or physical conditions preventing physical activity, and to be able to understand and complete all tests and questionnaires. Specific inclusion and exclusion criteria are shown in Table 1.

All participants were fully informed of any risk associated with the experiments before giving their written consent for participation. The study was approved by the ethics committee at Erasmus hospital (Université Libre de Bruxelles (ULB)) the 14 October 2024 (approval code: P2024/435/B4062024000314). Procedures were in accordance with the recommendations of the Helsinki Declaration. Subjects were randomly assigned by lottery into two groups: a moderate-intensity continuous training (MICT) group and a high-intensity interval training (HIIT) group.

Ultimately, the study was conducted on 25 participants. The difference of seven participants (two women and five men) between the initial and final samples is explained by two factors: some withdrew during the study due to injuries, illnesses, or personal reasons, while others were excluded from post-testing because they did not reach the minimum threshold of 80% training attendance.

### 2.2. Protocol

**Figure 1:**
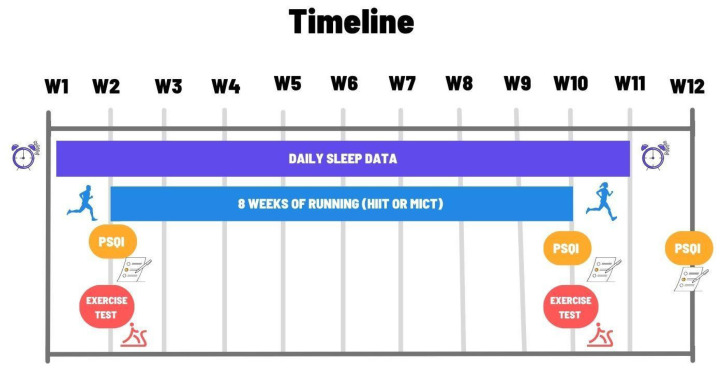
Summary of the timeline Between week 1 (W1) and week 12 (W12).

This study was conducted over a 12-week period (See Figure 1). Sleep data collection began one week prior to the start of training and the first cardiopulmonary exercise test (CPET), continued throughout the training period (8 weeks) [14], and extended 1 week post-training, totaling 10 weeks (See Figure 1, W1–W11). Upon selection, participants were registered on the “My CoachBox” platform, which enabled daily collection of sleep data and monitoring of training sessions.

A CPET on the treadmill (h/p/cosmos, sport and medical gmbh) was conducted during the second week (See Figure 1, W2), preceded by some anthropometric measurements (body height and body weight) and the determination of systolic and diastolic blood pressure. Additionally, all subjects completed the validated PSQI questionnaire at this timepoint to assess their sleep quality. This test battery was repeated after 8 weeks of training at W10, and the PSQI was repeated once more at W12 (see Figure 1).

#### 2.2.1. Cardiopulmonary Exercise Test (CPET)

The treadmill-based CPET followed a triangular protocol with three-minute stages (See Figure 1, W2 and W10) [15]. Speed increased progressively by 2 km/h for male participants and 1.5 km/h for female participants. The starting speed was set at 6 km/h for all participants, with a 1% incline applied from the outset to simulate outdoor air resistance [16].

At the end of each stage, several parameters were collected and recorded: perceived exertion using the modified Borg scale (0–10), capillary blood sampling from the fingertip to determine lactate values, and heart rate. The test continued until voluntary cessation by the participant, indicating an inability to sustain the effort.

Heart rate was monitored using a chest-mounted Polar device, and respiratory gas exchange was measured via a nose-and-mouth mask connected to the HypAir system. Capillary blood samples (10–20 μL) were analyzed using the EKF Biosen C-Line system to determine blood lactate concentration.

#### 2.2.2. Training

A personalized eight-week training program was implemented to assess the different impacts of HIIT and MICT modalities on physiological adaptations and sleep quality. Training protocols were based on those used in Pattyn et al. [14], with modifications to the MICT protocol in response to participant feedback and discussions from the referenced study. Target heart rate zones were set at 80% of the maximum heart rate in the MICT protocol. These adjustments resulted in a deviation from isocaloric balance between protocols. Hence, the estimated energy expenditure for each training session was approximately 273 kcal for HIIT and 352 kcal for MICT, based on the measurements reported by Pattyn et al. [14]. The training sessions were structured into specific heart rate windows to be maintained for defined durations.

During the first two weeks, each group completed two weekly sessions according to their assigned protocol: the HIIT group performed two interval training sessions, while the MICT group engaged in two continuous training sessions. Over the following six weeks, training frequency increased to three sessions per week. The MICT group continued with continuous training only, whereas the HIIT group alternated between two interval sessions and one continuous session identical to the MICT protocol. A minimum 24 h recovery interval between sessions was enforced to optimize recovery and prevent muscular fatigue.

The percentual HR values (%HR) used to structure these training intensities were individually determined for each participant during the initial cardiopulmonary exercise test (CPET) and lactate threshold determination, ensuring personalized and physiologically appropriate training loads.

**Figure 2:**
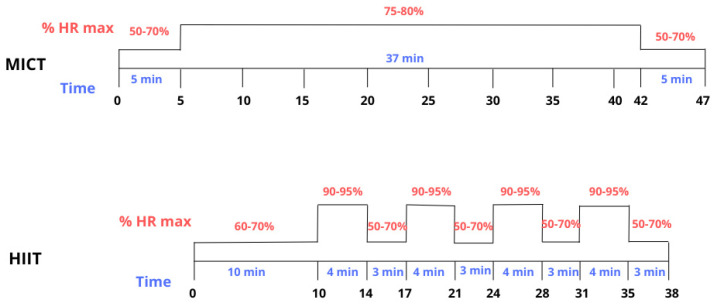
Plan of each training type [14]: Moderate Intensity Continuous Training (MICT) and High Intensity Interval Training (HIIT).

Each HIIT session began with a 10 min warm-up performed at 60–70% of the participant’s individual maximum heart rate (HRmax), followed by four interval cycles. High-intensity phases consisted of 4 min at 90–95% HRmax, while low-intensity recovery phases lasted 3 min at 50–70% HRmax (See Figure 2). Each MICT session started with a 5 min warm-up at 50–70% HRmax, followed by a continuous effort of 37 min maintained at 80–85% HRmax, and concluded with a 5 min cool-down at 50–70% HRmax (See Figure 2).

#### 2.2.3. Post-Training Test

The PSQI was reassessed on the day of the second exercise test (post-test) to evaluate changes in sleep quality specifically related to the eight-week training period (See Figure 1 W10). Anthropometric measurements and blood pressure were also re-evaluated prior to the test. The second CPET followed the same protocol as the initial test, but only participants who had completed at least 80% of training sessions were eligible. Finally, two weeks after the end of the training program, a final PSQI assessment was conducted to examine the persistence of training effects on sleep quality (See Figure 1 W12).

#### 2.2.4. Sleep and Training Data

A My Coach Box application (https://www.coachbox.app/, accessed on 22 November 2025) was used to plan, monitor, and analyze participants’ training sessions. Each subject was equipped with a smartwatch (Polar ignite 2) wearable compatible with the platform, allowing for automatic synchronization of training data. The interface enabled the recording and analysis of training metrics through specific graphs and indicators provided by the platform. This system ensured individualized tracking of performance and progression. Direct communication with participants was also possible, allowing for personalized recommendations, motivation and eventual adjustments. This approach optimized training adherence and ensured that each participant followed the defined protocol while receiving tailored support.

Daily monitoring of sleep parameters was conducted via My Coach Box platform, including both subjective sleep quality and sleep duration. Sleep quality was assessed each day by selecting one adjective from six options (very poor, poor, moderate, good, very good, excellent). These adjectives were converted into numerical values ranging from 0 to 10.

Sleep duration was recorded by entering the total number of hours slept. Alternatively, this measure could be derived from two simple questions: “What time did you go to bed last night?” and “What time did you wake up this morning?”

On the other hand, objective evaluation of sleep quality was performed using the Pittsburgh Sleep Quality Index (PSQI). This standardized tool consists of 19 items grouped into seven components, which together yield a global sleep quality score [15]. The subscales include: daytime sleepiness, sleep disturbances, sleep duration, sleep efficiency, sleep latency, use of sleep medication, and subjective sleep quality. The PSQI’s ease of use and its ability to differentiate between various sleep profiles make it a versatile clinical instrument.

### 2.3. Statistical Analysis

Initially, Shapiro–Wilk tests were conducted to validate the use of parametric statistical methods. The normality of data distribution was assessed and confirmed through these tests (*p* > 0.05). Variables such as maximum heart rate (HRmax), blood pressure, and threshold velocities did not meet the normality assumption, and for these parameters, non-parametric tests, specifically the Friedman test and the Mann–Whitney U test, were applied to examine the combined effects of time (pre–post) and group (HIIT vs. MICT) on these variables.

For all other variables, mixed-design repeated measures ANOVA was used to assess the interaction between intra-individual effects (pre–post) and inter-group effects (HIIT vs. MICT). This statistical approach was applied to analyze changes in BMI, PSQI scores, VO_2max_, and the percentages of VO_2max_ and heart rate at lactate thresholds before and after the 8-week training period, according to training modality.

Finally, two-tailed Pearson correlation tests were performed to evaluate the presence of linear relationships between two quantitative variables, namely, the change in PSQI score and the change in VO_2max_. Across all statistical analyses, a significance threshold of *p* < 0.05 was adopted.

## 3. Results

**Figure 3:**
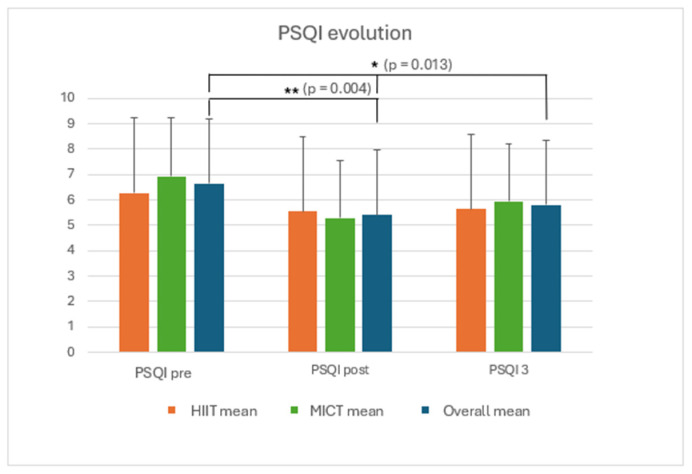
Evolution of the PSQI score (score/21) before and after the training and 2 weeks post-training; * *p* < 0.05, significant gap; ** *p* < 0.01, highly significant gap.

The participants’ characteristics are depicted in Table 2. Sleep quality (PSQI) improved significantly throughout the study period, which included 8 weeks of training and 2 weeks of retention (*p* = 0.013, η^2^ = 0.171, Cohens d = 1.88) independent of training type. Post hoc analyses are presented in Figure 3. No significant differences were found for sleep quality improvements between different exercise modalities (*p* = 0.809). The daily perceived sleep quality, reported through the training application, tended to improve (trend to significance) for all participants throughout the exercise regime (*p* = 0.063) with no differences between exercise modality (*p* = 0.544). No significant change in BMI nor blood pressure was observed after the training period or between the two training arms.

**Figure 4:**
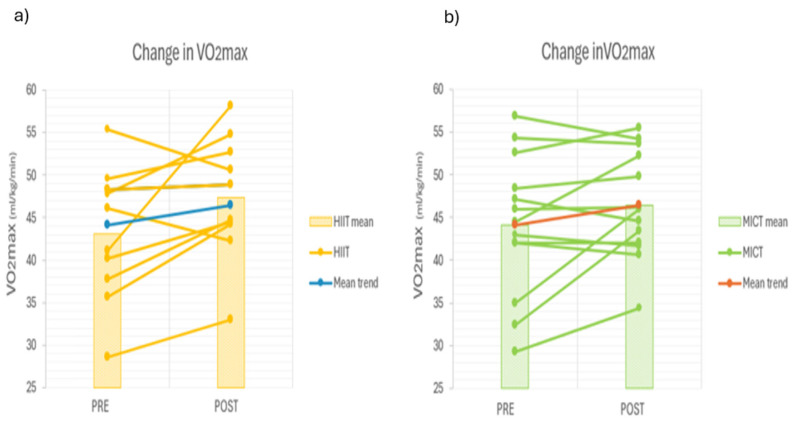
Individual evolution of the VO_2max_ between the pretest and the post-test for the MICT (**a**) and the HIIT (**b**) groups.

A statistically significant increase in VO_2max_ over time was observed for all participants (*p* = 0.009; η^2^ = 0.283, Cohen’s d = 5.92), with no significant interaction effect nor difference between the different exercise modalities. Individual VO_2max_ responses before and after MICT or HIIT training are presented in Figure 4. Additionally, maximal heart rate decreased significantly for both groups after 8 weeks of training (Cohen’s d = 3.64; HIIT: *p* = 0.003; MICT: *p* = 0.021) independent of training regime.

Both the aerobic (*p* < 0.001; η^2^ = 0.537, Cohen’s d = 12.6) and anaerobic lactate threshold (*p* = 0.002; η^2^ = 0.366, Cohen’s d = 10.3) shifted to a higher percentage of the average VO_2max_ after 8 weeks of training, regardless which training regime participants were assigned to, indicating a significant improvement of aerobic capacity after training. Similar results were found for the attained running speed, which increased significantly after 8 weeks of training at the aerobic (*p* = 0.003) and anaerobic threshold (*p* = 0.001). Post hoc analysis revealed, however, that the evolution of speed at the aerobic threshold was only significant for the MICT group (*p* = 0.014) but not for the HIIT group (*p* = 0.102). Nevertheless, no significant difference between groups was observed at the aerobic (*p* = 0.376) nor the anaerobic threshold (*p* = 0.879). With regard to the evolution of heart rate at the lactate thresholds, no significant differences were found over time, nor between groups.

**Figure 5:**
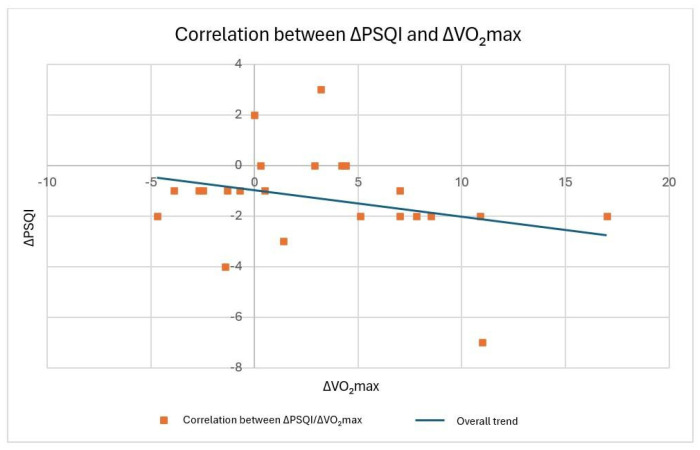
Relationship between the variation in the PSQI score and the evolution of the VO_2max_.

A weak and non-significant negative correlation (r = −0.295; *p* = 0.171) was found between the evolution of VO_2max_ and sleep quality (PSQI) after 8 weeks of training (see Figure 5).

Additionally, no significant associations were found between the evolution of speed or VO_2max_ at both the aerobic and anaerobic thresholds on the one hand and sleep quality on the other hand.

## 4. Discussion

The primary finding of this study is that eight weeks of thrice-weekly running sessions significantly improved sleep quality, irrespective of the training modality—whether moderate-intensity continuous training (MICT) or high-intensity interval training (HIIT) was imposed. No differential effects were observed between the two training regimes, suggesting that training intensity does not influence the trajectory of sleep quality improvements after 8 weeks. Secondly, VO_2max_ values as well as relative oxygen consumption and speed at the aerobic and anaerobic threshold significantly improved after 8 weeks of training, independent of which training regime was imposed. However, no significant association was found between the evolution of cardiorespiratory fitness and sleep quality after 8 weeks of training (MICT and HIIT). Regardless of the training protocol, consistent running appears to be a key driver of enhanced sleep and cardiorespiratory fitness outcomes.

The absence of a relationship between the improvement in VO_2max_ and PSQI may be due to the limited sample or the sensitivity of the PSQI tool to assess sleep quality. While the PSQI has been shown to be a valid and reliable tool to assess sleep quality, it of course lacks the capacity to profoundly measure sleep objectively [17,18].

Consistent with the findings of [13], the data collected in this study demonstrate a significant reduction in Pittsburgh Sleep Quality Index (PSQI) scores following an eight-week running intervention, indicating improved sleep quality. The reduction in PSQI score is significant but should be considered too low to have a meaningful clinical impact. Qin and colleagues [19] have calculated this based upon meta-analytical data in insomnia patients. However, this research has been performed in a different and rather clinical setting, and an improvement of one or two points is considered significant in terms of improving one’s daily functioning. This enhancement was evident in both training groups, suggesting that moderate-intensity continuous training (MICT) and high-intensity interval training (HIIT) exert comparable effects on sleep outcomes. Exercise is known to induce both physiological adaptation and fatigue due to increased energy demands and muscular exertion. Prior research has demonstrated that elevated energy expenditure throughout the day promotes deeper sleep phases, which are critical for physical and mental recovery [5]. Beyond energy expenditure, exercise influences biological regulators of sleep, such as adenosine accumulation—which facilitates sleep onset—and thermoregulation, where elevated body temperature during exertion followed by a post-exercise decline supports sleep initiation [4]. Additionally, the meta-analysis by De Nys et al. [13] put forward the importance of the link between cortisol regulation and sleep quality and indicated that physical exercise may positively influence both aspects. The observed improvements in sleep quality may thus be attributed to a combination of physiological and biochemical mechanisms. Regular exercise has been shown to modulate hormonal profiles, promote anti-inflammatory responses, enhance immune function, and improve sleep efficiency and duration [20]. Furthermore, exercise has long-term beneficial effects on body composition, basal metabolism, cardiac function, glycemic control, mood, and the immune system.

Given that HIIT and MICT activate distinct molecular pathways and elicit different physiological adaptations [21], it was interesting to observe an absence of significant differences in VO_2max_ and sleep outcomes. Previous studies have highlighted the superior effects of chronic HIIT on VO_2max_ and cardiovascular parameters [21,22,23]. This is predominantly due to an increased shear stress on the arteriolar wall, which induces superior arterial adaptations such as an increased NO bioavailability [23,24,25]. Metabolic or muscular adaptations such as mitochondrial remodeling, functioning or capillarization, on the contrary, seem to be more prevalent after a period of MICT instead of HIIT intervention [22,23,24]. It could be suggested that different mechanisms have led to similar VO_2max_ improvements within the MICT and HIIT groups of this study. Hence, it is well known that MICT is a potent stimulator of mitochondrial content in type I fibers [22,23,24,25,26] and improves metabolic efficiency as well, while HIIT improves mitochondrial content in type II fibers and has superior cardiovascular effects. This explanation can be supported by the fact that running speed at the aerobic threshold improved in the MICT cohort only, suggesting a higher aerobic power or velocity after 8 weeks of MICT. Central (cardiovascular) versus local (muscular) adaptations, which are more stimulated by HIIT and MICT, respectively, may explain the comparable VO_2max_ progression after 8 weeks. The weekly training sessions were conducted over an 8-week period, which may be considered relatively short to fully observe the physiological adaptations induced and listed before. However, the work of Murias et al. [27] shows that increases in VO_2max_ can already be detected after 3 weeks of a training program consisting of three cycle-ergometer sessions per week. Although notable progression continues to occur between weeks 8 and 12, measurements taken at week 8 are still sufficient to highlight meaningful cardiorespiratory improvements in the participants. The presented findings from this study underscore the importance of regular exercise, regardless of modality, as a potent, accessible, and non-pharmacological strategy for improving sleep quality. Healthcare professionals should consider incorporating structured physical activity into the holistic management of individuals with sleep disturbances or suboptimal sleep patterns.

This study has several limitations. First, the sample size in this study was quite low and could have impacted the interpretability of the results. The dropout of seven participants during the intervention particularly reduced the final sample size, potentially affecting statistical power. Further research on larger cohorts is needed to study the impact of these distinct exercise modalities on sleep quality and cardiorespiratory fitness. Second, the eight-week duration may be insufficient to capture long-term physiological adaptations such as mitochondrial biogenesis or increased capillary density. Nonetheless, Murias et al. [27] demonstrated that eight weeks of training can yield measurable improvements in VO_2max_, particularly when programs are individualized. It would be interesting to study what sleep quality evolutions could be observed after 12 weeks of different training modalities.

A third limitation to this study was the collection of sleep data solely through subjective sleep ratings, as well as the use of questionnaires only. Despite the fact that the PSQI has been shown to be a valid and reliable tool to assess sleep quality, it would have been interesting to assess differences before and after the training intervention through objectively measured polysomnography or in combination with actigraphy.

Future research should aim to extend the intervention period, increase the sample size, and incorporate a longer follow-up interval before the final PSQI assessment. Additionally, employing objective sleep measures such as polysomnography before and after different training modalities would provide deeper insights into the mechanisms underlying exercise-induced sleep improvements.

## 5. Conclusions

In conclusion, this study showed that exercise training improves sleep quality regardless of which training modality is imposed. Simply performing three 35–45 min running sessions per week for eight weeks leads to an improved aerobic and cardiorespiratory fitness, as well as improved sleep quality. Training intensity does not play a role in having superior effects on sleep quality, and no relation was found between cardiorespiratory fitness and sleep quality improvements. Physical activity thus appears to be an effective non-pharmacological strategy to improve sleep quality.

## Figures and Tables

**Table 1:** Inclusion and exclusion criteria.

Inclusion Criteria	Exclusion Criteria
Between 18 and 45 years old BMI between 18 and 30 Amateur athlete or sedentary individual No obligations significantly modifying sleep rhythm (night work, on-call duty) Alcohol consumption < 10 units/week Ability to perform and understand tests and questionnaires No chronic disease or health condition preventing the practice of physical activity	Chronic disease or health condition preventing the practice of physical activity <18 years old or >45 years old BMI < 18 or >30 High-level athlete (>8 h of training per week) Obligations significantly modifying sleep rhythm Alcohol consumption > 10 units/week Inability to perform or understand tests and questionnaires

**Table 2:** Participant characteristics and proportions.

Variables	Total	HIIT	MICT
Participants (*n*)	25	11	14
Average age (σ)	23 (±2)	23 (±2)	23 (±2)
Male, *n* (%)	16 (64%)	7 (64%)	9 (64%)
Female, *n* (%)	9 (36%)	4 (36%)	5 (36%)
Pre-average BMI (σ)	22.9 (±2)	23.6 (±2)	22.5 (±2)
Post-average BMI (σ)	22.9 (±2)	23.7 (±2)	22.2 (±2)
Pre-systolic BP (σ)	128 (±11)	129 (±14)	127 (±9)
Pre-diastolic BP (σ)	81 (±8)	82 (±7)	81 (±10)
Post-systolic BP (σ)	129 (±11)	127 (±14)	130 (±9)
Post-diastolic BP (σ)	80 (±8)	80 (±8)	80 (±8)

## Data Availability

The data presented in this study are available on request from the corresponding author. The data are not publicly available due to privacy or ethical restrictions.

## Abbreviations

The following abbreviations are used in this manuscript:

VO_2max_	Maximal Oxygen Consumption
MICT	Moderate-Intensity Continuous Training
HIIT	High-Intensity Interval Training
HR	Heart Rate
%HR	Percentage of Heart Rate
Hrmax	Maximal Heart Rate
BMI	Body Mass Index
CPET	Cardiopulmonary Exercise Test
PSQI	Pittsburgh Sleep Quality Index
